# Global and Local Persistence of Influenza A(H5N1) Virus

**DOI:** 10.3201/eid2008.130910

**Published:** 2014-08

**Authors:** Xianbin Li, Zhong Zhang, Ailian Yu, Simon Y. W. Ho, Michael J. Carr, Weimin Zheng, Yanzhou Zhang, Chaodong Zhu, Fumin Lei, Weifeng Shi

**Affiliations:** Chinese Academy of Sciences, Shenzhen, China (X. Li);; Taishan Medical College, Taian, Shandong, China (Z. Zhang, A. Yu, W. Shi);; Chinese Academy of Sciences, Beijing, China (X. Li, W. Zheng, Y. Zhang, C. Zhu, F. Lei);; University of Sydney, Sydney, New South Wales, Australia (S.Y.W. Ho);; University College Dublin, Dublin, Ireland (M.J. Carr); 1 These authors contributed equally to this article.

**Keywords:** H5N1, avian influenza, migration network, persistence, source, influenza, viruses

## Abstract

An understanding of the global migration dynamics of highly pathogenic avian influenza A(H5N1) virus is helpful for surveillance and disease prevention. To characterize the migration network of this virus, we used genetic analysis, which supported a global persistence model in which each of 9 regions acts to some extent as a source. Siberia is the major hub for the dispersal of the virus. Southeast Asia and Africa are major sources of genetically and antigenically novel strains. We found evidence of local persistence of the virus in Southeast Asia and Africa, which is rare for human influenza A viruses. The differences in migration dynamics between avian and human influenza viruses might help with the design of region-specific surveillance efforts and the selection of vaccine candidates.

Highly pathogenic avian influenza (HPAI) A(H5N1) virus is an ineradicable zoonotic virus that continues to mutate and reassort in nature and poses a serious threat to avian and human health. As the natural hosts of avian influenza viruses, wild birds are the main reservoir for the HPAI (H5N1) pandemic; whether these birds contribute to the viruses’ global circulation remains under debate (*1*–*3*).

Since their emergence in China in 1996 (*4*), HPAI (H5N1) viruses have spread to most Eurasian and African countries and have caused 650 laboratory-confirmed cases of human infection and 386 deaths (*5*). Understanding the migration dynamics of HPAI (H5N1) viruses is thus essential for surveillance and prevention of these infections in birds and humans and for policy decisions on vaccine development and/or implementation.

Numerous genetic studies have been conducted to determine the mechanisms underlying influenza A virus seasonality among humans; most results support a model of global migration (*6*–*11*). Rambaut et al. proposed a source–sink model for virus ecology (*7*), in which the tropics are the source regions and the subtropical and temperate zones of the Northern and Southern Hemispheres are the sink regions. Similarly, Russell et al. suggested that eastern and Southeast Asia comprise a regional circulation network that is the leading region for the evolution of human influenza viruses (*8*). However, Bedford et al. found that seasonal epidemics in the United States had seeded epidemics around the world in a pattern called global persistence (*9*). More recently, Bahl et al. found that the tropics (e.g., Southeast Asia and Hong Kong) did not maintain a source for annual epidemics of influenza A(H3N2) virus infection (*12*). Alternatively, each geographic region might act as a potential source, supporting the global persistence model.

Also extensively studied have been the migration mechanisms of avian influenza A(H5N1) virus (*13*–*15*). Despite the use of different methods, many studies reached the same conclusion: China is the source of multiple clusters of influenza A(H5N1) viruses identified from other countries in eastern and Southeast Asia (*13*–*18*). Liang et al. have also proposed that southern China and Southeast Asia might be the source of influenza A(H5N1) virus, seeding outbreaks elsewhere, and that eastern Siberia might be the source of influenza A(H5N1) virus cross-infection and genetic reassortment (*19*).

However, several questions remain with regard to the migration of HPAI (H5N1) viruses. For example, what are the features of their global migration network? Which region acts as the key node? Is southern China the only source of novel HPAI (H5N1) viruses? If not, what are the other sources? Are the sources stable, or do their contributions change with time?

To address these questions, we analyzed a large number of hemagglutinin gene sequences of influenza A(H5N1) viruses from avian hosts by using BEAST (*20*) and Migrate (*21*,*22*), which can estimate genetic diversity of each region and migration rates between regions. On the basis of these findings, we characterized the global migration network and studied the migratory mechanism of HPAI (H5N1) viruses.

## Materials and Methods

### Sequence Data and Genetic Diversity

All available sequences of the hemagglutinin gene of HPAI (H5N1) viruses isolated from avian hosts were obtained from Influenza Virus Resources at the National Center for Biotechnology Information (http://www.ncbi.nlm.nih.gov/genomes/FLU/). These sequences were aligned by using MUSCLE (*23*). After short sequences (>60 bp shorter than the full-length hemagglutinin-1) were removed, the final dataset included 3,365 sequences from 9 geographic regions, which, to our knowledge, made it the largest influenza A(H5N1) virus dataset analyzed (Table 1; Technical Appendix Figure 1). Therefore, we consider that the sequence data available in the database are informative and representative of the geographic distribution and global circulation of HPAI (H5N1) viruses, although they were not obtained through systematic global influenza virus sampling that was random in terms of time and space.

**Table 1:** Statistical analysis of geographic structure for highly pathogenic avian influenza A(H5N1) viruses*

Region	Maximum monophyletic clade size

Observed mean	95% CI	Null mean	95% CI	Significance, p value
South Korea and Japan	50.44	49.0–68.0	1.66	1.19–2.04	0.001
Siberia	8.90	8.0–11.0	1.59	1.11–2.01	0.001
Southeast Asia	311.54	296.0–440.0	4.57	3.93–5.95	0.001
Africa	47.73	38.0–64.0	3.55	3.12–4.30	0.001
Hong Kong	17.00	17.0–17.0	1.99	1.65–2.24	0.001
China	32.88	28.0–42.0	3.74	3.23–4.65	0.001
Europe	80.08	80.0–80.0	2.21	2.01–3.00	0.001
Central and western Asia	5.23	3.0–8.0	1.32	1.00–2.00	0.001
Southern Asia	106.00	106.0–106.0	2.03	1.75–2.30	0.001

*Analyzed by using Bayesian Analysis of Time Series (*27*).

To evaluate whether the classification of 9 regions was appropriate, we used the same method for estimating nucleotide diversity of avian influenza A(H5N1) virus that had been used for influenza A(H3N2) virus (*9*). Within-region nucleotide diversity was estimated in terms of π_w ._

where n is the number of regions and refers to diversity estimates in which both samples in each pair are from region *i*.

The overall between-region diversity was estimated as π_b._

where π^(^*^i,j^*^)^ refers to diversity estimates in which 1 sample is from region *i* and the other sample is from region *j*.

Confidence intervals were estimated by taking 1,000 bootstrap replicates from the total pool of sequences. *F_ST_* (genetic distance) was calculated as (π_b_−π_w_)/π_b_, with *F_ST_* >0 indicating genetic isolation among regions (*24*) and supporting the geographic classification mentioned above.

### Estimating Global Parameters and Testing Geographic Association at Tips

The program Migrate requires input of 2 parameters: the transition/transversion ratio (κ) and the rate of nucleotide substitution (μ). We estimated these parameters by using the Bayesian phylogenetic method implemented in BEAST version 1.7.2 (*20*). For all analyses, we used the uncorrelated lognormal relaxed molecular clock to accommodate rate variation among lineages (*25*). We used the HKY85 model (*26*) of nucleotide substitution to parameterize the mutational process; equilibrium nucleotide frequencies were derived from observed frequencies; equilibrium nucleotide frequency rates were homogeneous across sites. Posterior distributions of parameters were estimated by using Markov chain Monte Carlo (MCMC) sampling. Samples were drawn every 5,000 steps over a total of 4.0 × 10^7^ steps, and 2.5 × 10^7^ steps were removed as burn-in. The transition/transversion ratio (κ) was estimated to be 9.163 (95% CI 8.633–9.676). The rate of nucleotide substitution () was estimated to be 5.595 × 10^−3^ substitutions/site/year (95% CI 5.249 × 10^−3^– 5.970 × 10^−3^ substitutions/site/year).

To estimate the extent of geographic structure (extent to which viruses from the same geographic region are more likely to cluster together in the phylogenetic tree than expected by chance) in the HPAI (H5N1) influenza virus populations, we performed a phylogenetic-trait association analysis on the posterior distribution of trees produced by BEAST. These geographic regions were coded onto the tips of the 3,000 trees sampled from the posterior, which were then analyzed by using the maximum monophyletic clade size statistic implemented in the Bayesian Analysis of Time Series program with 1,000 randomizations (*27*). For each of the 9 regions included in the analysis, the Bayesian Analysis of Time Series program was used to calculate a p value, which indicated whether the sequences from this region are more inclined to cluster together in the tree than expected by chance.

### Estimating Migration Rates between Regions through Resampling

To estimate coalescent parameters for each geographic region, we used an MCMC technique implemented in Migrate version 3.3.0 (*21*,*22*). The prior distribution of Θ (mutation-scaled population size) and 2 Nm (migration rate) values was assumed to be exponential with a mean of 1, and mutational parameters were fixed in the analyses. To minimize the influence of potential sampling biases on our results, we performed independent analyses of 100 resampled replicates. For each replicate, we randomly sampled 50 sequences without replacement from each region (Technical Appendix Table 2). For each of the 100 bootstrap replicates, 50 MCMC simulations were run for 6 × 10^6^ steps each. The first 5 × 10^6^ steps of each chain were removed as burn-in. Parameter values were sampled every 10^4^ steps. Convergence was assessed visually and through comparison of chains by using the Gelman-Rubin convergence statistic (*28*). We combined the remaining samples from each chain to give a total of 5,000 samples for each of the resampled replicates. Estimates of migration rates varied little across the 100 replicates (Technical Appendix Table 1); mean values are shown in Table 2.

**Table 2:** Means and 95% credible intervals of the total immigration and emigration rates for highly pathogenic avian influenza A(H5N1) viruses across resampled replicates and trunk proportion of each region, 1996–2012*

Region	Mean (95% credible interval)		
	Immigration	Emigration	Trunk proportion
Europe	1.24 (0.61–2.25)	0.89 (0.49–1.58)	0.01 (0.00–0.05)
Hong Kong	0.65 (0.34–1.13)	0.67 (0.41–1.05)	0.06 (0.04–0.12)
Africa	0.26 (0.18–0.38)	0.98 (0.62–1.45)	0.25 (0.23–0.29)
China	0.46 (0.25–0.81)	0.69 (0.41–1.08)	0.24 (0.17–0.31)
Central and western Asia	1.95 (1.18–2.95)	0.71 (0.40–1.24)	0.01 (0.00–0.03)
Southern Asia	0.40 (0.28–0.60)	0.56 (0.32–0.93)	0.02 (0.00–0.10)
Southeast Asia	0.25 (0.16–0.38)	0.37 (0.23–0.56)	0.38 (0.35–0.41)
South Korea and Japan	1.05 (0.52–2.72)	1.03 (0.36–2.60)	0.00 (0.00–0.02)
Siberia	1.45 (0.57–3.35)	1.81 (0.84–4.05)	0.04 (0.00–0.11)

*Migration rates are given in migration events per lineage per year.

### Genealogical Inference and Trunk Extraction

Most of the viruses we analyzed were isolated from domestic chickens and ducks. To infer the genealogy, we reduced the dataset so that a maximum of 30 sequences per year were sampled from chickens and ducks from each region. Combining this subset of sequences with those from the other hosts yielded a final dataset of 2,392 sequences; this dataset had notably fewer sequences from Africa, China, and Southeast Asia than did the original dataset (Technical Appendix Table 2). We fixed Θ and 2Nm at the values estimated in the previous analysis.

We ran 4 MCMC chains for 2 × 10^8^ steps each, of which the first 10^8^ steps were removed as burn-in; genealogies were sampled every 10^5^ steps. We combined the remaining samples of 4,000 genealogical trees and performed trunk reconstruction on them.

Trunk extraction and processing was performed by using the program PACT (http://www.trevorbedford.com/pact), which is able to estimate the mean and 95% credible interval for the proportion of the trunk assigned to each geographic region (*9*). The bigger the proportion, the more the corresponding region accounts for virus variation and evolution. By calculating the proportion of sampled genealogies for which the trunk is assigned to a particular region at different points in time, we could also assess the temporal dynamics, which illustrate the annual change of trunk proportion for each region.

### Testing the Robustness of the Results

For many sequences, the time of isolation is known to the nearest year only, which limits the precision of estimates of relative genetic diversity through time. Therefore, we further analyzed a subsample in which we included only sequences isolated during 2006–2011 and for which detailed isolation times were available. This dataset included 1,173 samples from 6 geographic regions. We repeated the above analyses on this subset of the dataset. Our sampling strategies can be found in Technical Appendix Table 3. MCMC simulations were run for 4 × 10^7^ steps, and 2 × 10^7^ steps were removed as burn-in.

### Estimating Genetic Diversity of Each Region

To estimate the relative genetic diversity of each region through time, we extracted sequences of viruses from each region from the subsampled subdataset with 1,173 sequences from 2006 through 2011, which composed 5 new datasets: for Africa, Southeast Asia, China, southern Asia, and Europe (including Siberia). Each of these datasets was analyzed by using the Bayesian skyride method (*29*) in BEAST. Because the sizes of these subdatasets differed, we ran MCMC simulations for different steps for each and collected samples every 10^4^ steps.

## Results

### Geographic Structure

Our dataset included 3,365 HPAI (H5N1) virus hemagglutinin gene sequences exclusively identified from nonmammalian hosts. On the basis of geography and sampling density, we classified these sequences into 9 regions: China (mainland China, n = 768), Hong Kong Special Administrative Region (n = 168), South Korea and Japan (n = 105), Siberia (n = 95), Southeast Asia (n = 1,024), southern Asia (n = 176), western and central Asia (n = 64), Europe (n = 261), and Africa (n = 704). This geographic classification is significantly supported by a phylogenetic trait–association test; all p values were <0.001, indicating that the sequences are more inclined to cluster together by geographic location than would be expected by chance (Table 1). Moreover, on average, genetic diversity between regions, π_b_ = 42.64 × 10^−3^ (95% CI 41.09 × 10^−3^–48.81 × 10^−3^), is greater than within regions, π_w_ = 17.21 × 10^−3^ (95% CI 16.33 × 10^−3^–18.19 × 10^−3^). *F_ST_* was estimated to be 0.596 (95% CI 0.573−0.649), indicating genetic isolation among regions and also supporting the rationale for classifying the HPAI (H5N1) virus hemagglutinin sequences into 9 regions (*24*). Further information about regional genetic diversity is provided in Technical Appendix Table 4.

### Global Migration and Persistence Indicated by Migration Rates

**Figure 1:**
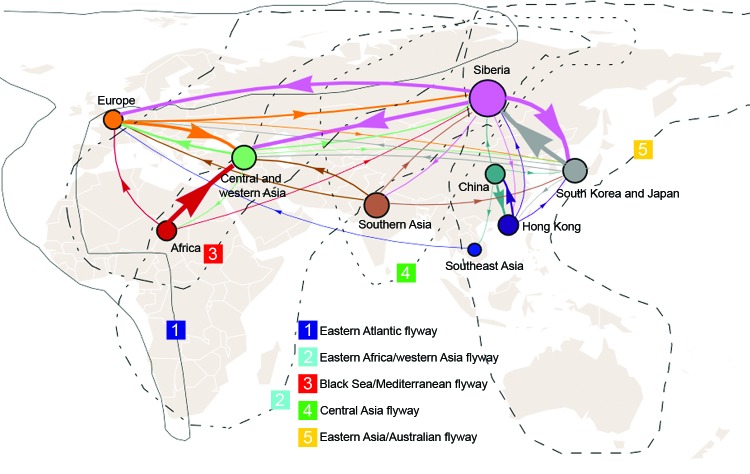
Global migration patterns of highly pathogenic avian influenza A(H5N1) viruses estimated from sequence data sampled during 1996–2012. Arrows represent direction of movement, and arrow width is proportional to the migration rate. Migration rates <0.07 migration events per lineage per year are not shown. The area of each circle is proportional to the region’s eigenvector centrality; larger circles indicate crucial nodes in the migration network**.**

We used the sampling strategy of Bedford et al. to study the global migration of human influenza A(H3N2) virus (*9*). Our results support a global migration model (Figure 1; Table 2) in which all regions in the analysis are connected to form a global migration network; the migration rate between any 2 regions is >0 (Technical Appendix Table 1). However, the geographic regions play different roles in the connectivity of the global migration network, as indicated by the varying circle sizes in Figure 1; all 9 regions act to some extent as sources. Specifically, migration rates between temperate regions >0 indicate that 1 temperate region could seed epidemics in other temperate regions. Therefore, these results support a model for the global persistence of HPAI (H5N1) virus (*9*,*12*). 

In detail, this network contains 4 notable features (Figure 1; Table 2). First, Siberia is the most active node; the immigration rate is the second highest, and the emigration rate is the highest. High rates of migration are found for Siberia to South Korea and Japan, western and central Asia, and Europe. Second, for central and western Asia, the immigration rate is very high; the 3 major sources are Africa, Siberia, and Europe. Third, for Southeast Asia and Africa, immigration rates are relatively low (0.25 and 0.26, respectively), and for Africa and China, immigration rates are lower than emigration rates. Fourth, for South Korea and Japan, immigration and emigration rates are high. Pairwise immigration and emigration rates are given in Technical Appendix Table 1.

### Genealogical History and Persistence of HPAI (H5N1) Viruses

We next inferred the genealogical history of the HPAI (H5N1) virus population. As described in previous studies, a human influenza virus tree can be characterized by a long trunk and short side branches (*9*,*30*). In brief, the trunk of a genealogical tree is composed of progenitor strains whose mutations are maintained (*9*). Therefore, strains located along the trunk account for a greater amount of virus evolution and genetic variation than do the strains on the side branches.

**Figure 2:**
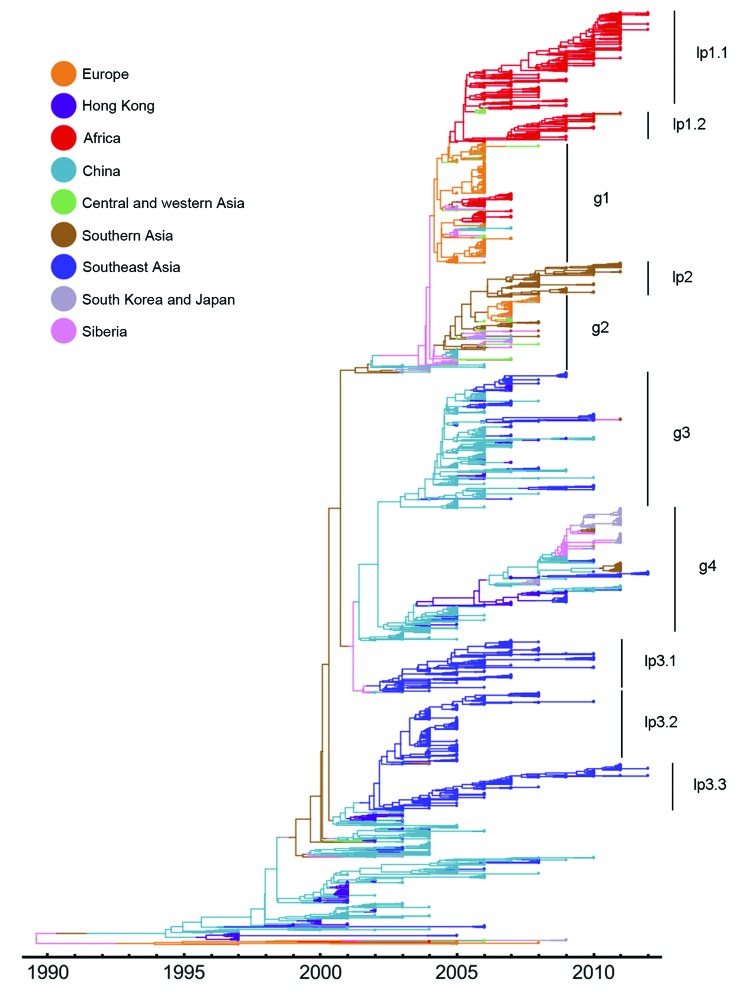
Estimated genealogy of 3,365 highly pathogenic avian influenza A(H5N1) viruses sampled during 1996–2012. The maximum a posteriori tree was estimated by using Migrate version 3.3.0 (*21**,**22*). Each tip represents a virus sequence. Colors indicate the sampling region, either actual (tips) or estimated (branches).

Consistent with our findings described above, the reconstructed genealogical history supports a global migration model because several lineages consist of virus strains from different geographic regions (e.g., g1–g4 in Figure 2). However, after viruses of these lineages emerge, they spread to other geographic regions, and generally these viruses persist over years through migration (e.g., g2–g4 in Figure 2). Therefore, the genealogy of HPAI (H5N1) viruses also supports a model of global persistence.

In contrast with human influenza A/H3N2 virus phylogenies, the HPAI (H5N1) influenza virus tree shows several long side branches (Figure 2), which supports a model of local persistence (lp) (*9*,*30*,*31*). These viruses on the side branches reside in Southeast Asia (lp3.1 to lp3.3), Africa (lp1.1 and lp1.2), and southern Asia (lp2). Of particular note, these lineages can persist over extended periods. For example, a lineage from Southeast Asia persisted from around 2002 to 2012 (lp3.3). Local persistence of HPAI (H5N1) virus has led to the co-circulation of different lineages in Southeast Asia during 2002–2010.

### Trunk Proportion for Different Regions

Using a structured coalescent approach, we calculated the proportion of the trunk assigned to each geographic region (Table 2). Generally, a higher trunk proportion implies that the corresponding region accounts for more virus variation and evolution and that that region is more likely to be the source of the virus. In contrast, despite previous evidence that China was the region containing the influenza source population for HPAI (H5N1) viruses (*13*–*18*), only 24% (95% CI 17%–31%) of the trunk of the genealogical tree is assigned to China. Instead, Southeast Asia occupies the largest proportion of the trunk, 38% (95% CI 35%–41%), and the contribution of Africa to the trunk is 25% (95% CI 23%–29%). Although Hong Kong has a robust surveillance and reporting system for cases of HPAI virus infection, it accounts for a comparatively low level of the trunk of the influenza A(H5N1) virus genealogy, 6% (95% CI 4%–12%).

**Figure 3:**
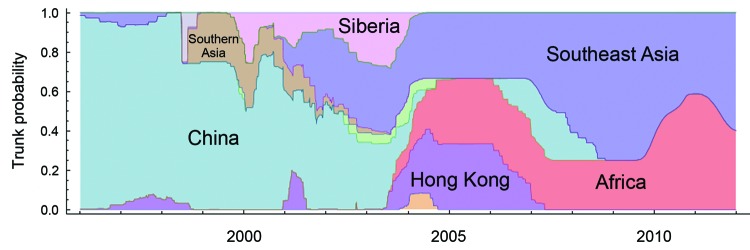
Temporal changes in geographic regions along the trunk of the highly pathogenic avian influenza A(H5N1) virus genealogical tree.

Further analysis of the trunk proportion reveals a dynamic change of regions over time (Figure 3), suggesting that the contribution of each geographic region to virus variation and evolution changes annually. Before 2004, China was the source for most of the evolution and variation of HPAI (H5N1) viruses. After 2001, however, Southeast Asia began to act as another primary source for novel strains. Since 2004, viruses on the trunk have resided less in China and more in Southeast Asian and African countries.

### Genetic Diversity of HPAI (H5N1) Viruses from Different Regions

**Figure 4:**
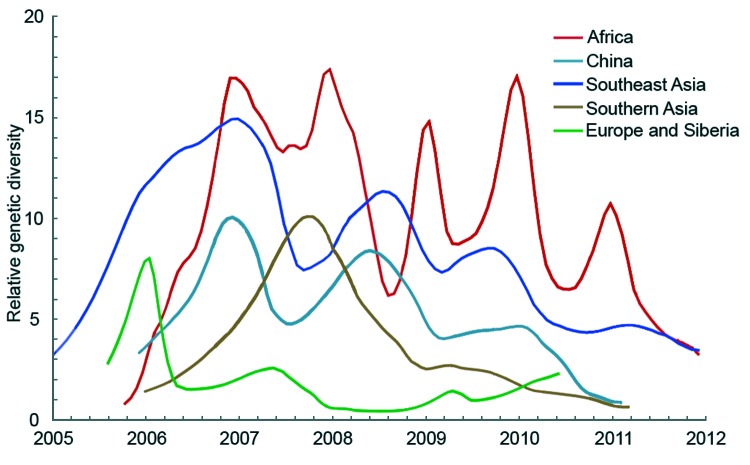
Bayesian skyride median of relative genetic diversity of highly pathogenic avian influenza (H5N1) virus in each region, 2006–2011. Shading represents winter (October–March) in the Northern Hemisphere**.**

Because only regions with higher genetic diversity are most likely to be the virus source, we calculated the genetic diversity of 5 geographic regions by using sequences from 2006 through 2011 for which month of isolation was known. In this analysis, Europe and Siberia were combined because *F_ST_* = 0, indicating a lack of genetic isolation between them (*24*). Although Africa, Southeast Asia, and China show influenza seasonality to different degrees, genetic diversities of HPAI (H5N1) viruses from these 3 regions are higher than those of the remaining regions throughout the year (Figure 4). Specifically, virus genetic diversity is highest in Africa and second highest in Southeast Asia; both regions maintain a relatively high level of diversity throughout the year. Furthermore, virus genetic diversity in Africa shows a clear seasonal pattern of change; peaks occur during the Northern Hemisphere winter. Activity of HPAI (H5N1) virus in Southeast Asia and China typically, but not always, peaks during the Northern Hemisphere winter (Figure 4).

## Discussion

Our large-scale genetic analysis of HPAI (H5N1) viruses supports a global persistence model in which each region acts to some extent as a source. Siberia seems to play a vital role in this migration network, connecting Europe, central and western Asia, and South Korea and Japan. This finding is consistent with the fact that multiple bird migration flyways intersect in Siberia; the region is also one of the most commonly used breeding sites and in summer contains a high number of wild birds (*19*).

Although it has not been widely acknowledged that migratory birds act as vectors for the spread of HPAI (H5N1) viruses in Eurasia and Africa (*32*–*34*), it is intriguing that the migration network described here approximates the major flyways of migratory birds. This finding coincides with evidence obtained by comparing the spatiotemporal characteristics of wild bird migration and influenza A(H5N1)virus outbreaks, especially along the central Asia flyway (*19*,*35*–*37*).

Southern China has been regarded as the source of HPAI (H5N1) viruses (*13*–*18*). However, our results show that China is not the only source, although our data support the view that China was the major center for the evolution and variation of HPAI (H5N1) virus before 2004. The data also indicate that the tropics (Southeast Asia and Africa) have been the major sources since 2004. The role of these regions as genetic reservoirs for subtype H5N1 viruses is underscored by the higher genetic diversities of viruses in these regions compared with those of other regions. Therefore, increased sampling from Southeast Asia and Africa is vital for understanding the global dynamics of HPAI (H5N1) viruses.

With regard to emergence of novel virus variants, our results also support a source–sink model for HPAI (H5N1) virus, as described for human influenza A virus subtypes H1N1 and H3N2 (*7*), in which the tropics are the source regions and the Northern and Southern Hemispheres are the sink regions. This finding is not inconsistent with the global migration pattern of HPAI (H5N1) virus because China, as referred to here, is largely represented by tropical/subtropical southern China (*13*–*16*). In addition, most (93%) of the trunk of the genealogical tree is in the tropics and China (including Hong Kong), and the remaining regions, such as Siberia, play major roles in migration and genetic reassortment and are less responsible for emergence of variants with novel hemagglutinin proteins.

The relative genetic diversities of the viruses in Africa, Southeast Asia, and China change regularly over time, but peaks do not always appear concurrently among regions. Generally, peaks of influenza virus activity in Africa appear in winter, whereas those of Southeast Asia and China sometimes appear in other seasons. This finding can potentially be explained by the fact that influenza A(H5N1) virus has become endemic to Southeast Asia and China, where >70% of domestic ducks are raised. Domestic ducks can asymptomatically shed high titers of subtype H5N1 virus for several days (*38*).

The influenza A(H5N1) virus genealogical tree notably contains long side branches; some lineages (mostly comprising hemagglutinin sequences from Southeast Asia, Africa, and southern Asia) persist for years. These phylogenies support a model of local persistence of HPAI (H5N1) viruses. Specially, local persistence has led to co-circulation of multiple lineages and is likely to confound efforts to control the spread and selection of HPAI (H5N1) virus vaccine candidates. However, these patterns contrast with those described for seasonal human influenza A(H3N2) virus, for which global persistence plays a much larger role in the migration network (*9*,*12*).

Likewise, for human influenza A viruses, mutations on side branches have limited effects with regard to producing antigenically novel variants. This limitation is because mutations on side branches experience genetic bottlenecks and are quickly lost, such as at the end of peak influenza season (*9*,*30*). For HPAI (H5N1) viruses, however, mutations on side branches can have influential effects, because these mutations will be maintained for years because of local and/or global virus persistence.

The main study results were obtained by using the sampling year of the sequences. To evaluate effects of this potential bias on our results, we repeated the analyses by using a subsample of our dataset with sequences for which year and month of collection were known. Results from this subsample are broadly consistent with those from the whole dataset. Therefore, use of only the sampling year plays a limited role in the results. Detailed information about this subsample and the results of our additional analyses are provided in the Technical Appendix Figures 2–5 and Tables 3 and 5–8.

In conclusion, we characterized the major features of the HPAI (H5N1) virus migration network and found evidence to support global and local persistence of this virus. We also drew attention to the role of Southeast Asia and Africa as genetic reservoirs in the origins of genetically and antigenically novel influenza A(H5N1) virus variants, which has, to our knowledge, been previously underestimated. Our results call for reassessment of the role of each geographic region in the migration network and in the genetic source of HPAI (H5N1) viruses and suggest that region-specific surveillance policies and vaccine candidate selection strategies should be considered.

Technical Appendix Data used to demonstrate global and local persistence of influenza A(H5N1) virus.
