# Supplementary material for: Global and Local Persistence of Influenza A(H5N1) Virus
Source: Emerg Infect Dis. 2014 Aug;20(8):1287–95. doi: 10.3201/eid2008.130910 (PMC4111173; doi:10.3201/eid2008.130910)
Supplement: Technical Appendix — Data used to demonstrate global and local persistence of influenza A(H5N1) virus. [file 13-0910-Techapp-s1.pdf]

Article DOI: http://dx.doi.org/10.3201/eid2008.130910

Global and Local Persistence of Influenza A(H5N1) Virus

Technical Appendix

Technical Appendix Table 1. Estimates for immigration (columns) and emigration (rows) rates between each pair of regions measured in terms of migration events per lineage per year

	Europe	Hong Kong	Africa	China	Central & West Asia	South Asia	Southeast Asia	South Korea & Japan	Siberia
Europe	-	.04 (0.02, 0.07)	.04 (0.03, 0.06)	.02 (0.01, 0.04)	.41 (0.24, 0.61)	.04 (0.03, 0.06)	.02 (0.01, 0.03)	.08 (0.05, 0.15)	.24 (0.09, 0.57)
Hong Kong	.06 (0.04, 0.10)	-	.02 (0.01, 0.02)	.25 (0.13, 0.42)	.06 (0.05, 0.08)	.03 (0.03, 0.05)	.06 (0.04, 0.10)	.09 (0.06, 0.15)	.09 (0.06, 0.13)
Africa	.14 (0.07, 0.25)	.03 (0.02, 0.05)	-	.02 (0.01, 0.03)	.58 (0.38, 0.80)	.04 (0.02, 0.06)	.01 (0.01, 0.02)	.06 (0.05, 0.10)	.09 (0.05, 0.14)
China	.06 (0.04, 0.10)	.29 (0.14, 0.51)	.02 (0.01, 0.02)	-	.06 (0.05, 0.08)	.04 (0.03, 0.05)	.08 (0.05, 0.12)	.07 (0.05, 0.11)	.07 (0.05, 0.11)
Central & West Asia	.27 (0.13, 0.51)	.04 (0.03, 0.06)	.07 (0.04, 0.13)	.02 (0.02, 0.03)	-	.06 (0.04, 0.08)	.02 (0.01, 0.02)	.08 (0.06, 0.12)	.16 (0.09, 0.29)
South Asia	.08 (0.05, 0.12)	.04 (0.03, 0.06)	.02 (0.02, 0.03)	.02 (0.01, 0.04)	.17 (0.09, 0.30)	-	.02 (0.01, 0.02)	.07 (0.05, 0.10)	.13 (0.06, 0.25)
Southeast Asia	.07 (0.04, 0.11)	.06 (0.03, 0.10)	.02 (0.01, 0.02)	.03 (0.02, 0.06)	.05 (0.04, 0.07)	.03 (0.02, 0.04)	-	.05 (0.04, 0.08)	.05 (0.04, 0.07)
South Korea & Japan	.10 (0.04, 0.21)	.07 (0.03, 0.15)	.02 (0.02, 0.03)	.03 (0.02, 0.06)	.12 (0.07, 0.24)	.05 (0.03, 0.09)	.02 (0.01, 0.03)	-	.61 (0.13, 0.79)
Siberia	.45 (0.20, 0.84)	.08 (0.04, 0.14)	.05 (0.03, 0.06)	.06 (0.03, 0.13)	.49 (0.26, 0.76)	.11 (0.07, 0.18)	.02 (0.02, 0.03)	.54 (0.18, 0.91)	-

Technical Appendix Table 2. Number of sequences used from each geographic region in different stages of the analysis

	Diversity estimates	Equal sampling	Genealogical estimates
Europe	261	50	261
Hong Kong	168	50	168
Africa	704	50	376
China	768	50	576
Central & West Asia	64	50	64
South Asia	176	50	153
Southeast Asia	1024	50	594
South Korea & Japan	105	50	105
Siberia	95	50	95
Total	3365	450	2392

Technical Appendix Table 3. Number of sequences used from each geographic region in different stages of the analysis using the sub-datasets with sequences from 2006 to 2011*

	Diversity estimates	Equal sampling	Genealogical estimates
Europe	101	55	101
Africa	534	55	534
China	70	55	70
South Asia	84	55	84
Southeast Asia	329	55	329
Siberia	55	55	55
Total	1173	330	1173

*Sequences isolated from this period but without month information available are removed from this analysis.

Technical Appendix Table 4. Regional genetic diversity π arrayed below the diagonal, measured in terms of 10^{-3} substitutions per site, and regional F_{ST} arrayed above diagonal, with 95% confidence intervals determined by 1,000 bootstrap replicates

	Europe	Hong Kong	Africa	China	Central & West Asia	South Asia	Southeast Asia	South Korea & Japan	Siberia
Europe	8.2 (7.5, 9.1)	0.75 (0.73, 0.84)	0.07 (–0.05, 0.70)	0.60 (0.57, 0.76)	0.20 (0.16, 0.45)	–0.18 (–0.48, 0.37)	0.62 (0.58, 0.74)	0.71 (0.65, 0.91)	0.62 (0.54, 0.91)
Hong Kong	47.1 (44.6, 70.6)	14.7 (13.0, 16.7)	0.70 (0.68, 0.72)	0.03 (–0.06, 0.11)	0.65 (0.60, 0.70)	0.65 (0.61, 0.69)	0.47 (0.43, 0.52)	0.77 (0.72, 0.80)	0.79 (0.77, 0.82)
Africa	15.4 (12.5, 44.9)	54.4 (52.1, 56.7)	18.2 (16.4, 20.0)	0.53 (0.50, 0.56)	0.06 (0.00, 0.12)	0.50 (0.46, 0.55)	0.59 (0.57, 0.62)	0.85 (0.84, 0.87)	0.77 (0.74, 0.79)
China	46.6 (43.1, 76.4)	22.1 (19.7, 24.5)	49.3 (46.5, 52.3)	28.1 (24.8, 31.0)	0.51 (0.46, 0.55)	0.49 (0.43 0.55)	0.24 (0.19, 0.29)	0.64 (0.60, 0.68)	0.61 (0.57, 0.64)
Central & West Asia	18.1 (14.2, 27.3)	50.5 (48.9, 52.5)	20.5 (18.1, 23.3)	49.0 (46.3, 51.7)	20.4 (15.1, 26.5)	0.13 (–0.01, 0.26)	0.50 (0.45, 0.55)	0.78 (0.71, 0.83)	0.28 (0.18, 0.38)
South Asia	15.0 (12.0, 27.0)	58.3 (55.3, 60.9)	44.6 (41.2, 48.2)	53.2 (48.8, 58.4)	26.8 (23.1, 31.0)	26.2 (22.6, 30.3)	0.55 (0.51, 0.59)	0.72 (0.68, 0.76)	0.69 (0.63, 0.75)
Southeast Asia	52.0 (48.8, 74.8)	43.6 (40.7, 46.7)	60.9 (58.7, 63.4)	38.9 (37.5, 40.5)	52.1 (50.5, 53.9)	64.0 (60.8, 67.3)	31.3 (28.4, 34.3)	0.70 (0.65, 0.74)	0.63 (0.60, 0.66)
South Korea & Japan	23.2 (17.7, 70.6)	39.9 (34.3, 45.9)	74.9 (70.5, 78.8)	44.3 (41.3, 47.2)	55.2 (45.3, 64.1)	53.7 (48.4, 59.2)	58.6 (50.2, 67.1)	3.9 (3.7, 4.2)	0.89 (0.79, 0.93)
Siberia	18.3 (13.0, 70.0)	45.3 (42.0, 48.5)	47.8 (41.8, 53.6)	40.6 (38.6, 42.6)	16.9 (14.5, 19.7)	49.4 (42.5, 56.0)	47.3 (45.7, 49.4)	37.2 (19.0, 56.2)	3.9 (3.4, 4.5)

Technical Appendix Table 5. Statistical analysis of geographic structuring for HPAI (H5N1) viruses from 2006 to 2011 using BaTS

Statistic	observed mean	95% CI	null mean	95% CI	significance
MC (Siberia)	8.99	7.0–16.0	1.56	1.06–2.02	0.001
MC (Africa)	437.66	431.0–440.0	5.62	4.55–7.49	0.001
MC (Southeast Asia)	189.78	188.0–188.0	3.68	3.11–4.97	0.001
MC (South Asia)	56.00	56.0–56.0	1.92	1.42–2.29	0.001
MC (Europe)	32.09	32.0–32.0	2.05	1.70–2.55	0.001
MC (China)	8.84	7.0–13.0	1.77	1.26–2.11	0.001

Technical Appendix Table 6. Means and 95% confidence intervals of the total immigration and emigration rates across resampled replicates from 2006 to 2011*

	Immigration	Emigration
Europe	1.12 (0.77, 1.61)	0.33 (0.23, 0.54)
Africa	0.13 (0.10, 0.19)	0.52 (0.35, 0.75)
China	0.20 (0.14, 0.27)	0.55 (0.45, 0.72)
South Asia	0.40 (0.31, 0.51)	0.46 (0.29, 0.66)
Southeast Asia	0.14 (0.10, 0.20)	0.28 (0.22, 0.38)
Siberia	0.95 (0.68, 1.39)	0.82 (0.58, 1.10)

*The migration rates are measured in terms of migration events per lineage per year.

Technical Appendix Table 7. Estimates for immigration (columns) and emigration (rows) rates between each pair of regions measured in terms of migration events per lineage per year using sequences from 2006 to 2011

	Europe	Africa	China	South Asia	Southeast Asia	Siberia
Europe	-	.04 (0.03, 0.07)	.02 (0.02, 0.02)	.06 (0.04, 0.07)	.02 (0.01, 0.03)	.19 (0.13, 0.35)
Africa	.31 (0.21, 0.48)	-	.02 (0.01, 0.02)	.06 (0.04, 0.08)	.01 (0.01, 0.02)	.11 (0.08, 0.16)
China	.13 (0.10, 0.16)	.02 (0.02, 0.02)	-	.04 (0.03, 0.05)	.07 (0.05, 0.09)	.30 (0.25, 0.40)
South Asia	.14 (0.10, 0.22)	.02 (0.02, 0.03)	.02 (0.02, 0.03)	-	.02 (0.01, 0.02)	.25 (0.13, 0.36)
Southeast Asia	.10 (0.08, 0.13)	.02 (0.02, 0.02)	.03 (0.02, 0.05)	.04 (0.03, 0.05)	-	.10 (0.08, 0.13)
Siberia	.44 (0.28, 0.63)	.03 (0.02, 0.04)	.12 (0.08, 0.15)	.20 (0.17, 0.25)	.03 (0.02, 0.04)	-

Technical Appendix Table 8. Means and 95% credible intervals over sampled genealogies for the location of the genealogy trunk between the years of 2006 and 2011

	Trunk proportion
Europe	0.01 (0.00, 0.04)
Africa	0.22 (0.19, 0.27)
China	0.32 (0.18, 0.52)
South Asia	0.00 (0.00, 0.02)
Southeast Asia	0.43 (0.27, 0.57)
Siberia	0.02 (0.00, 0.06)

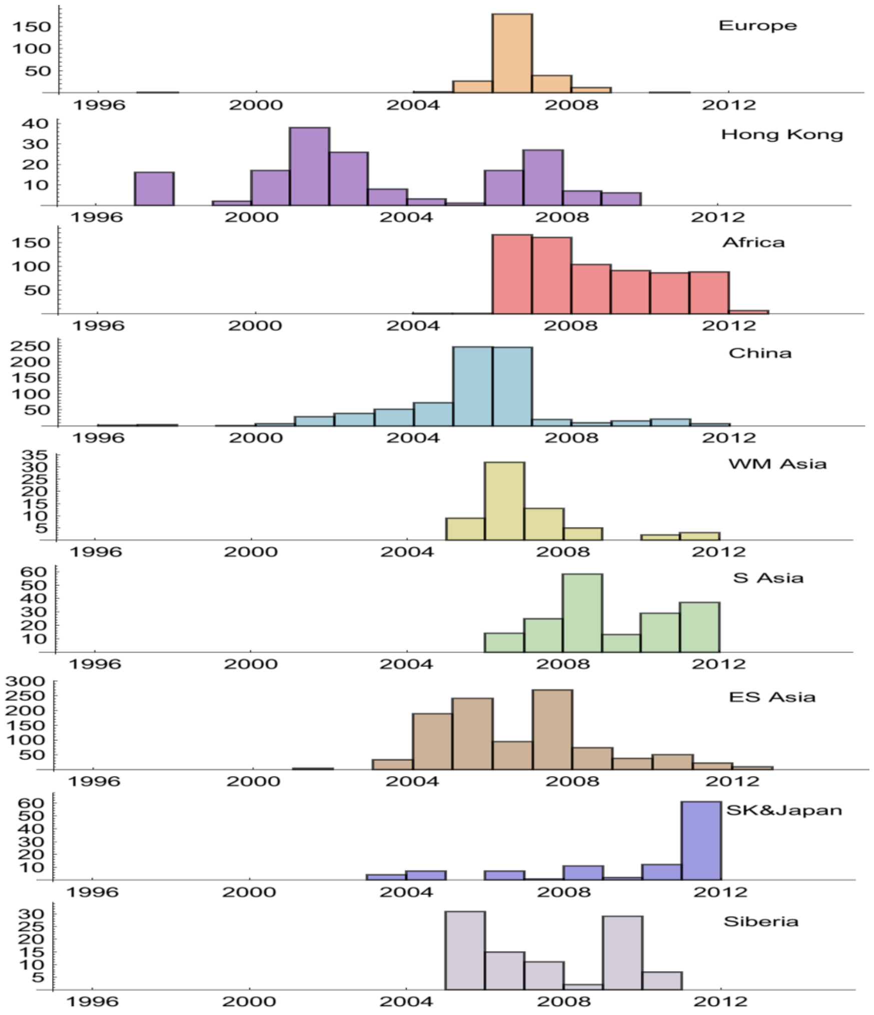

Technical Appendix Figure 1. Temporal and geographic distribution of 3,365 hemagglutinin gene sequences from 1996 to 2011.

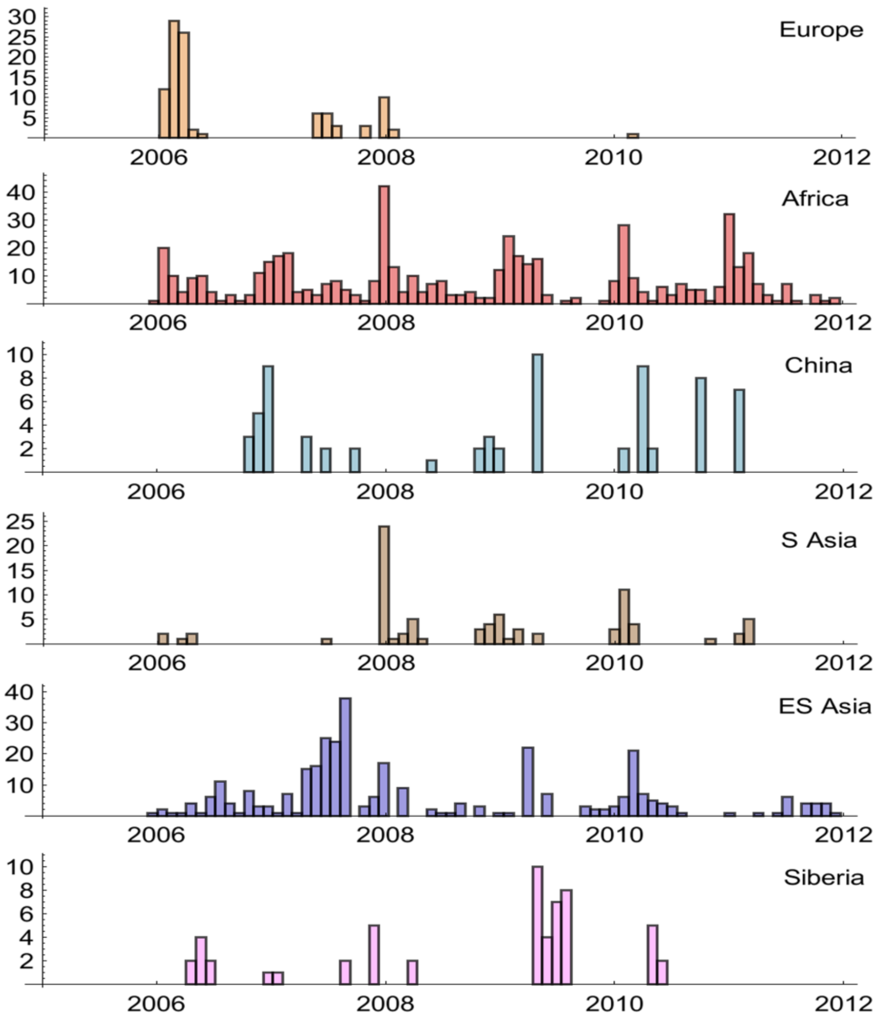

Technical Appendix Figure 2. Monthly and geographic distribution of sequences from 2006 to 2011.

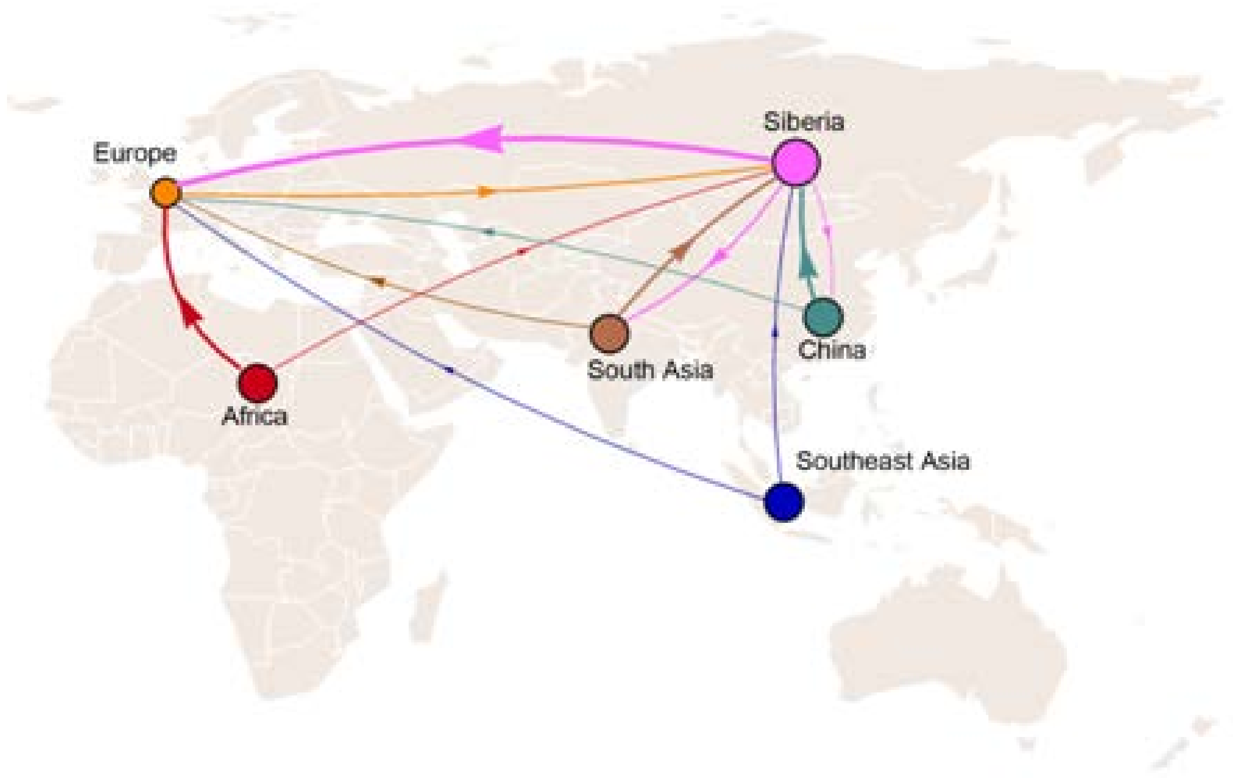

Technical Appendix Figure 3. Global migration patterns of HPAI (H5N1) viruses estimated from sequence data between 2006 and 2011. Arrows represent movement direction and arrow width is proportional to the migration rate. Arrows with migration rate smaller than 0.1 migration events per lineage per year are not shown. Circle areas are proportional to a region's eigenvector centrality. The larger a circle is, the greater the importance of the node in the migration network.

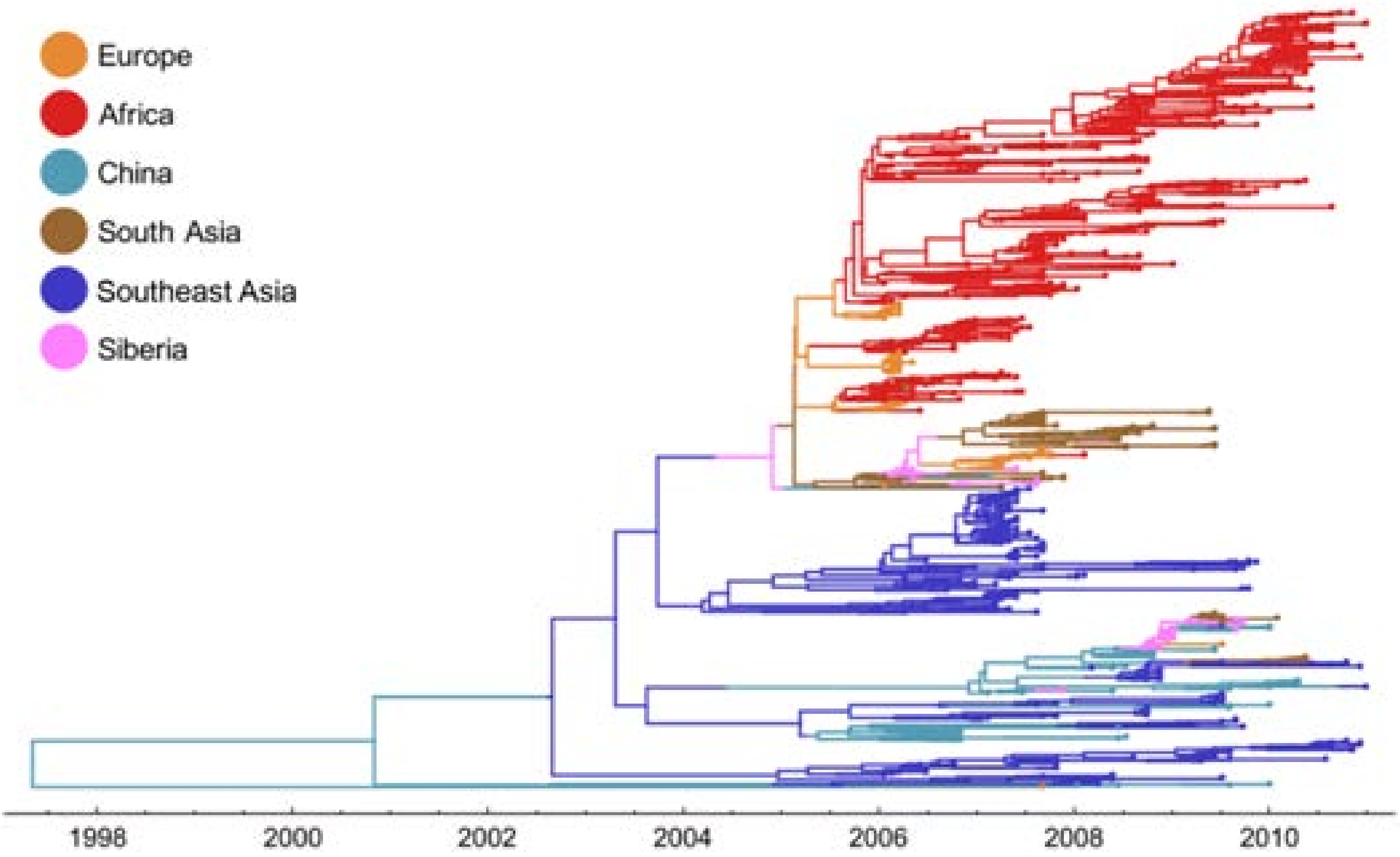

Technical Appendix Figure 4. Genealogy of 1,173 HPAI (H5N1) viruses sampled from 2006 to 2011. This tree was generated using Migrate v3.3.0 and has the highest posterior probability. Each point represents a virus sequence and color indicates the sampling region of this sequence.

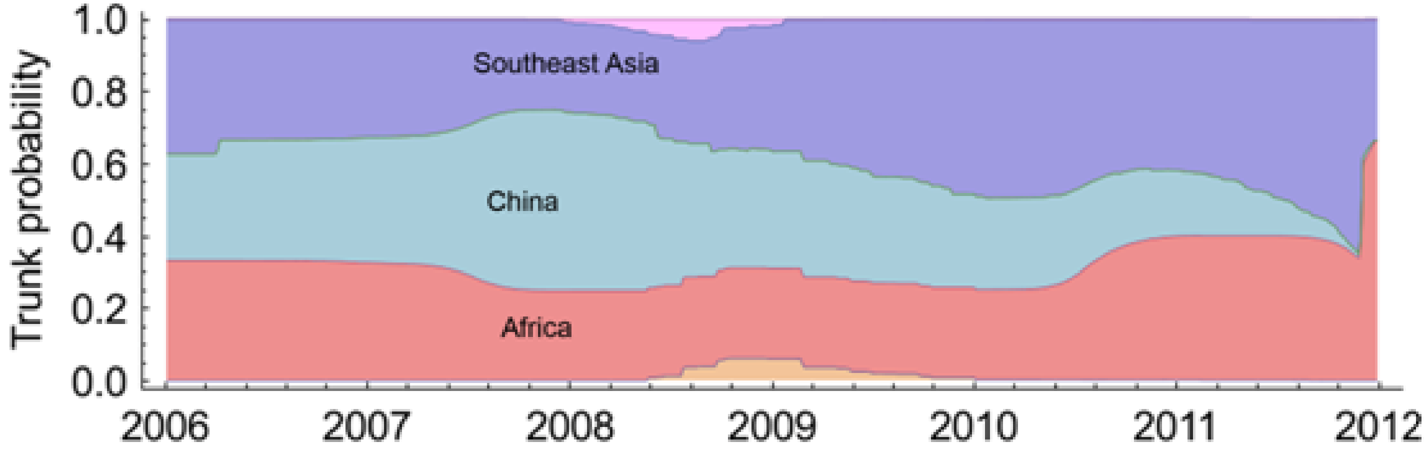

Technical Appendix Figure 5. Temporal changes of geographic region along the trunk of the HPAI (H5N1) tree from 2006 to 2011
